# Advanced ANN-LMB modeling of hepatitis B transmission across sexual networks and its disability burden

**DOI:** 10.1038/s41598-025-31252-2

**Published:** 2025-12-18

**Authors:** Rahat Zarin, Kamel Guedri, Basim M. Makhdoum, Hatoon A. Niyazi, Bandar M. Fadhl, Hamiden Abd El-Wahed Khalifa

**Affiliations:** 1 Department of Mathematics, Faculty of Science, King Mongkut’s University of Technology, Thonburi (KMUTT), Bangkok, 10140 Thailand; 2 Mechanical Engineering Department, College of Engineering and Architecture, Umm Al-Qura University, P. O. Box 5555, 21955 Mecca, Saudi Arabia; 3 Department of Clinical Microbiology and Immunology, Faculty of Medicine, King Abdulaziz University, 21589 Jeddah, Saudi Arabia; 4 King Salman Center for Disability Research, 11614 Riyadh, Saudi Arabia; 5 Department of Operations and Management Research, Faculty of Graduate Studies for Statistical Research, Cairo University, Giza, 12613 Egypt

**Keywords:** Parameter sensitivity, Artificial neural networks, Sexual transmission, Disability modeling, Epidemiological forecasting, Computational biology and bioinformatics, Diseases, Mathematics and computing

## Abstract

Hepatitis B virus (HBV) remains a major global health concern, with sexual transmission being a key driver among adults. This study develops a gender-stratified compartmental model of HBV spread that integrates long-term disability through gender-specific parameters. A key contribution is the integration of mechanistic modeling with artificial neural networks (ANNs), enabling efficient emulation of the model’s nonlinear dynamics. Numerical solutions from the classical Runge-Kutta 4th-order (RK4) method were used as training data for an ANN optimized with the Levenberg–Marquardt algorithm (ANN–LMB). The trained ANN accurately reproduces compartmental dynamics with minimal error and provides a fast surrogate for sensitivity exploration. Analysis of the basic reproduction number ($R_0$) reveals the strong influence of same-sex transmission rates, contact patterns, and disability onset parameters. These findings highlight the importance of behavioral interventions, vaccination coverage, and early detection of chronic carriers. Overall, the proposed ANN–LMB framework enhances computational efficiency and offers a biologically informed approach for exploring complex HBV transmission dynamics.

## Introduction

Hepatitis B virus (HBV) remains a major global health challenge, affecting over two billion individuals worldwide and causing nearly 900,000 deaths annually^1,2^. The virus primarily targets the liver, leading to acute and chronic infections that can result in cirrhosis and hepatocellular carcinoma. HBV is transmitted through contact with infected blood, semen, or other body fluids, with sexual contact being a major route among adults^3–5^. High-risk behaviors, such as unprotected intercourse and multiple sexual partners, particularly among chronically infected individuals, significantly contribute to HBV spread. The virus is estimated to be 50–100 times more infectious than HIV and exhibits greater environmental stability than hepatitis C virus^6,7^. Despite effective vaccines and antiviral therapies, HBV persists as a serious public health concern due to incomplete immunization coverage, behavioral risk factors, and limited access to early diagnosis and treatment^8–10^. While antiviral therapy can suppress viral replication, a complete cure for chronic HBV infection remains elusive^11^. Hence, prevention through vaccination, awareness, and behavioral modification remains central to HBV control^1^. Sexual transmission, both heterosexual and homosexual, continues to drive HBV prevalence among adults. Heterosexual contact remains the dominant route, while male-to-male transmission accounts for a significant proportion of new infections, particularly in urban and high-risk populations^4,9,12^. Although less common, evidence suggests that female-to-female transmission can occur through close sexual contact and shared partners^13–15^. These behavioral differences underscore the importance of gender-stratified modeling approaches for understanding HBV transmission dynamics.

A robust body of epidemiological evidence demonstrates that chronic hepatitis B virus infection is a substantial contributor to population-level disability, as quantified by years lived with disability (YLDs) and disability-adjusted life years (DALYs). The Global Burden of Disease Study 2019 estimated that approximately 316 million individuals were chronically infected with HBV, resulting in 555000 deaths in that year; these deaths reflect only part of the health impact, as HBV also causes extended morbidity due to cirrhosis and hepatocellular carcinoma (HCC)^16,17^. The DALY metric, which combines premature mortality with time lived in a disabled state, integrates the non-fatal consequences of HBV infection such as prolonged hepatic dysfunction, fatigue, and cognitive impairment^18^. In 2019, an estimated 34.5%, of age-standardized HBV DALYs were attributable to modifiable risk factors (alcohol, tobacco, high BMI), suggesting an interplay between HBV progression and lifestyle-related disability^19^. This highlights the potential for prevention strategies to reduce long-term disability outcomes among carriers. Geographically, HBV-related disability burden is unequally distributed; low and middle-income regions exhibit higher DALY rates, paralleling lower access to vaccination and treatment services^16,20^. Moreover, studies from countries like Brazil and Iran show specific YLD contributions from HBV-associated cirrhosis. For example, in Brazil in 2008, chronic HBV and its complications caused approximately 57380 DALYs (5.5 YLDs per 100000), with most of the burden arising from premature death but with a measurable YLD component^21^. Finally, attributing HBV burden to high-risk groups reveals further insights: among people who inject drugs, about 1.1,% of HBV-related DALYs were linked directly to injection exposure in 2013, underscoring how behavioral risk factors amplify disability risk in vulnerable subpopulations^22^. Taken together, these findings underscore that chronic HBV not only leads to increased mortality but also results in measurable disability at population scale. For modelling purposes, parameters reflecting YLDs and DALYs enable the translation of carrier prevalence into projected disability burden, guiding planning for health services and disability-focused interventions.

Various mathematical frameworks have been developed to investigate the transmission dynamics and control strategies of HBV, reflecting decades of global research efforts^23–25^. For example, age-structured and control-based modeling approaches have been applied to examine HBV transmission in specific populations, such as those in New Zealand^26^. The effectiveness of vaccination as a primary control measure has been analyzed in several studies^27,28^, while multi-group and cost-effectiveness formulations have provided further insights into the impact of intervention programs^29,30^. Other models have incorporated time delays and migration effects to capture temporal and spatial heterogeneity in HBV dynamics^31–33^. Although these traditional compartmental models have significantly advanced the understanding of HBV spread, they are often limited in their ability to represent nonlinear memory effects and parameter uncertainty inherent in biological systems. Recent developments have sought to address these challenges through advanced computational paradigms, such as stochastic solvers and artificial neural networks. For instance, Anwar et al^34^. introduced a stochastic Runge–Kutta approach for HBV modeling, while related studies extended adaptive neural architectures to computer virus propagation^35^ and nonlinear measles transmission^36^. Further advancements in autoregressive neural solvers and hybrid intelligent frameworks have been reported for multi-delay and biomedical dynamical systems^37,38^. These contributions highlight the growing relevance of AI-assisted numerical modeling in infectious disease research and motivate the present study’s use of the ANN–LMB framework for HBV dynamics, incorporating disability progression and gender-specific transmission mechanisms.

Artificial Neural Networks are increasingly used in infectious disease research because they can approximate highly nonlinear interactions among biological, social, and environmental factors that influence disease dynamics. Their data-driven nature allows them to learn from epidemiological observations, such as temporal incidence patterns, demographic distributions, climatic variables, and clinical indicators, without the need to specify detailed mechanistic equations^39^. This flexibility makes ANN-based approaches particularly effective when data are incomplete, noisy, or uncertain. To strengthen biological interpretability, recent frameworks have combined neural computation with traditional compartmental modeling concepts. Rodríguez et al^40^. proposed the concept of Epidemiologically-Informed Neural Networks (EINNs), which integrate SIR and SEIR-type dynamics within neural layers, while Rodríguez et al^41^. expanded this approach through Graph Neural Networks (GNNs) that capture spatial, social, and mobility-based interactions. In malaria studies, ANN architectures have been adapted for both ecological prediction and diagnostic applications.Rajnarayanan et al^42^. introduced a hybrid structure linking temperature- and altitude-dependent malaria transmission to a combination of ANN, CNN, and RNN models for outbreak risk estimation. Similarly, Guedri et al^43^. demonstrated that coupling stochastic solvers with ANNs enhances the accuracy and robustness of malaria forecasts under uncertainty, illustrating the strength of hybrid computational techniques in epidemiology. Jalloh et al^44^. further reported that optimized ANN architectures achieved mean absolute percentage errors below 5% for malaria incidence prediction in Sierra Leone outperforming conventional regression-based forecasting tools. In diagnostic applications, Soner et al^45^. developed an ANN–CNN–RNN ensemble for automated malaria detection in microscopic images, reaching over 97% classification accuracy. The success of these methods highlights the versatility of ANN-driven modeling in representing disease transmission, guiding surveillance, and improving diagnostic efficiency in endemic regions. Beyond infectious diseases, ANN-based systems have been increasingly utilized across biomedical and environmental domains, including neural activity modeling^46^, immune and inflammatory responses^47^, aquatic and ecological systems^48^, cancer risk prediction^49^, and tumor therapy modeling^50^. Together, these advances demonstrate that neural computation offers a flexible, accurate, and generalizable framework for studying nonlinear processes in biological and health sciences.

A key novelty of this study lies in the integration of HBV-induced disability into a transmission model that accounts for both heterosexual and homosexual dynamics. By introducing gender-specific disability probabilities ($\theta _f$, $\theta _m$) and onset rates ($\delta$) directly into the compartmental structure, the model captures long-term functional impairments resulting from chronic HBV infection without increasing structural complexity. Furthermore, this study explores the application of ANNs to enhance the modeling of HBV transmission and progression. The proposed framework combines data-driven learning with mechanistic disease modeling to ensure both computational efficiency and high predictive accuracy. At the core of the methodology is a system of nonlinear ODEs that describe HBV dynamics across seven compartments. These ODEs are numerically solved using the classical RK4 method to produce high-fidelity solution trajectories. These trajectories are then used as reference data for supervised ANN training. The network is trained using the ANN–LMB, which enables rapid convergence and accurate prediction of compartmental behaviors, including susceptible, infective, carrier, and recovered populations. Several recent studies have applied ANN techniques to complex epidemiological systems. For example, Guedri et al^51^. developed an ANN-based model for Ebola transmission that incorporates delay effects and neurological complications. Zarin^52^ investigated influenza dynamics using a spatially diffused SVEIR model coupled with neural networks. Sabir and colleagues further demonstrated the effectiveness of Meyer wavelet-based^53^ and fractional-order neuro-evolutionary solvers^54,55^ in modeling nonlinear and singular epidemic systems. In a related effort, Guedri et al^56^. modeled syphilis progression by integrating ANN frameworks to simulate disability risks within compartmental structures. Building upon these foundations, the present study extends ANN–LMB methodologies to HBV modeling with a specific focus on long-term disability impacts, sexual transmission heterogeneity, and gender-specific health burdens.

The main contributions of this study are summarized as follows:

- Formulation of a nonlinear, gender-stratified HBV transmission model that captures both sexual (heterosexual and homosexual) and non-sexual transmission pathways.
- Incorporation of long-term disability effects among carrier populations using fractional disability parameters and progression rates.
- Use of RK4-based numerical solutions as training targets for supervised ANN learning.
- Implementation of the ANN architecture with the Levenberg–Marquardt optimization algorithm to achieve fast convergence and accurate compartmental approximation.
- Validation of the ANN–LMB framework using mean squared error (MSE), error histograms, and performance evaluation plots.

## Model formulation

This study focuses on the adult population, which is divided into four main epidemiological compartments: susceptible, infective, carrier, and recovered individuals. To capture gender-specific dynamics, the susceptible group is subdivided into female susceptibles, denoted by $\textbf{S}_f(t)$, and male susceptibles, denoted by $\textbf{S}_m(t)$. Similarly, the infective population is divided into female infectives, $\textbf{I}_f(t)$, and male infectives, $\textbf{I}_m(t)$. The carrier class is separated into female carriers, $\textbf{C}_f(t)$, and male carriers, $\textbf{C}_m(t)$. The recovered individuals are collectively represented by $\textbf{R}(t)$^57^. Accordingly, the total population $\textbf{N}(t)$ at time *t* is given by:

$$\textbf{N}(t) = \textbf{S}_f(t) + \textbf{I}_f(t) + \textbf{C}_f(t) + \textbf{S}_m(t) + \textbf{I}_m(t) + \textbf{C}_m(t) + \textbf{R}(t).$$

The model assumes that recovered individuals acquire permanent immunity, with no subsequent loss of protection. Migration (both immigration and emigration) is ignored, and population renewal occurs only through births. The recruitment rates of females and males are represented by $A_f$ and $A_m$, respectively. Two transmission mechanisms are considered: non-sexual and sexual transmission. Sexual transmission is further divided into heterosexual and homosexual interactions. The non-sexual transmission rate is denoted by $\lambda$, while the sexual transmission rates are expressed as $\lambda _{mf}$, $\lambda _{fm}$, $\lambda _{ff}$, and $\lambda _{mm}$, corresponding to the following interactions:

- $\lambda _{mf}$: transmission from males to females,
- $\lambda _{fm}$: transmission from females to males,
- $\lambda _{ff}$: transmission among females,
- $\lambda _{mm}$: transmission among males.

Following the formulation proposed by Mclean and Blumberg^58^, these transmission rates are defined as:

1 $$\begin{aligned} \begin{aligned}&\lambda _{fm} = \frac{2 \beta _{fm} c_{fm}\left( \textbf{I}_f + \epsilon \textbf{C}_f\right) }{N}, \\&\lambda _{mf} = \frac{2 \beta _{mf} c_{mf}\left( \textbf{I}_m + \epsilon \textbf{C}_m\right) }{N}, \\&\lambda _{mm} = \frac{2 \beta _{mm} c_{mm}\left( \textbf{I}_m + \epsilon \textbf{C}_m\right) }{N}, \\&\lambda _{ff} = \frac{2 \beta _{ff} c_{ff}\left( \textbf{I}_f + \epsilon \textbf{C}_f\right) }{N}, \\&\lambda = \beta \left( \textbf{I}_m + \textbf{I}_f\right) + \epsilon \beta \left( \textbf{C}_m + \textbf{C}_f\right) . \end{aligned} \end{aligned}$$

In these expressions, $\beta _{fm}$, $\beta _{mf}$, $\beta _{ff}$, and $\beta _{mm}$ denote the corresponding transmission coefficients, while $c_{fm}$, $c_{mf}$, $c_{ff}$, and $c_{mm}$ represent the average contact rates. The parameter $\epsilon$ accounts for the reduced infectiousness of carrier individuals compared with acutely infected individuals. The HBV model developed in^57^ provides the basis for this study; however, it does not include the long-term disability outcomes associated with chronic hepatitis B infection. To improve its biological relevance and public health applicability, the model is extended to incorporate disability dynamics among carriers. Disability due to chronic HBV infection often results from liver-related complications that impair physical function, cause fatigue, and reduce cognitive capacity. To represent these effects, two parameters are introduced: $\theta _f$ and $\theta _m$, representing the proportions of female and male carriers who develop disabling complications, respectively. A new rate parameter, $\delta$, describes the average rate at which disability becomes clinically significant among carriers. The resulting extended model, which includes both traditional infection dynamics and disability progression, is described by the following system of nonlinear differential equations:

2 $$\begin{aligned} \left\{ \begin{aligned}&\frac{\textrm{d} \textbf{S}_f}{\textrm{d} t}=A_f - (\lambda + \mu _0 + \gamma _3) \textbf{S}_f - a \left( \frac{2 \beta _{mf} c_{mf}(\textbf{I}_m + \epsilon \textbf{C}_m)}{\textbf{N}}\right) \textbf{S}_f - (1-a) \left( \frac{2 \beta _{ff} c_{ff}(\textbf{I}_f + \epsilon \textbf{C}_f)}{\textbf{N}}\right) \textbf{S}_f, \\&\frac{\textrm{d} \textbf{I}_f}{\textrm{d} t}=\lambda \textbf{S}_f + a \left( \frac{2 \beta _{mf} c_{mf}(\textbf{I}_m + \epsilon \textbf{C}_m)}{\textbf{N}}\right) \textbf{S}_f + (1-a) \left( \frac{2 \beta _{ff} c_{ff}(\textbf{I}_f + \epsilon \textbf{C}_f)}{\textbf{N}}\right) \textbf{S}_f - (\mu _0 + \gamma _1) \textbf{I}_f, \\&\frac{\textrm{d} \textbf{C}_f}{\textrm{d} t}=q_f \gamma _1 \textbf{I}_f - (\mu _0 + \mu _1 + \gamma _2 + \theta _f \delta ) \textbf{C}_f,\\&\frac{\textrm{d} \textbf{S}_m}{\textrm{d} t}=A_m - (\lambda + \mu _0 + \gamma _3) \textbf{S}_m - b \left( \frac{2 \beta _{fm} c_{fm}(\textbf{I}_f + \epsilon \textbf{C}_f)}{\textbf{N}}\right) \textbf{S}_m - (1-b) \left( \frac{2 \beta _{mm} c_{mm}(\textbf{I}_m + \epsilon \textbf{C}_m)}{\textbf{N}}\right) \textbf{S}_m, \\&\frac{\textrm{d} \textbf{I}_m}{\textrm{d} t}=\lambda \textbf{S}_m + b \left( \frac{2 \beta _{fm} c_{fm}(\textbf{I}_f + \epsilon \textbf{C}_f)}{\textbf{N}}\right) \textbf{S}_m + (1-b) \left( \frac{2 \beta _{mm} c_{mm}(\textbf{I}_m + \epsilon \textbf{C}_m)}{\textbf{N}}\right) \textbf{S}_m - (\mu _0 + \gamma _1) \textbf{I}_m, \\&\frac{\textrm{d} \textbf{C}_m}{\textrm{d} t}=q_m \gamma _1 \textbf{I}_m - (\mu _0 + \mu _1 + \gamma _2 + \theta _m \delta ) \textbf{C}_m, \\&\frac{\textrm{d} \textbf{R}}{\textrm{d} t}=\gamma _3(\textbf{S}_f + \textbf{S}_m) + (1 - q_f) \gamma _1 \textbf{I}_f + (1 - q_m) \gamma _1 \textbf{I}_m + \gamma _2(\textbf{C}_f + \textbf{C}_m) - \mu _0 \textbf{R}. \end{aligned} \right. \end{aligned}$$

The corresponding initial conditions are:

$$\begin{aligned} \textbf{S}_f(0) = \textbf{S}_f^0, \quad \textbf{I}_f(0) = \textbf{I}_f^0, \quad \textbf{I}_m(0) = \textbf{I}_m^0, \quad \textbf{S}_m(0) = \textbf{S}_m^0, \quad \textbf{C}_f(0) = \textbf{C}_f^0, \quad \textbf{C}_m(0) = \textbf{C}_m^0, \quad \textbf{R}(0) = \textbf{R}^0 \ge 0. \end{aligned}$$

**Biological interpretation of disability and recovery parameters** The parameters $\theta _f$ and $\theta _m$, together with $\delta$, represent the transition of a subset of carriers into long-term disability states resulting from HBV-related complications such as cirrhosis and hepatocellular carcinoma. These individuals are assumed to be non-infectious and are therefore removed from the active transmission dynamics. The term $\gamma _2$ accounts for the rare functional recovery observed clinically in a small proportion of chronic HBV cases, corresponding to spontaneous clearance of HBsAg. This formulation allows the model to capture both the epidemiologically inactive disabled population and the rare but biologically possible recovery pathway without increasing structural complexity. The numerical values of the parameters are given in Table 1.

**Table 1:** Model parameters and values.

Parameter	Value	Comments	Source
*a*	0.75	The proportion of heterosex in female susceptibles	^57^
*b*	0.75	The proportion of heterosex in male susceptibles	^57^
$A_f$	$8,304,000 \ \text {year}^{-1}$	Recruitment rate of females	^59^
$A_m$	$8,304,000 \ \text {year}^{-1}$	Recruitment rate of males	^59^
$\mu _0$	$6.9 \times 10^{-3} \ \text {year}^{-1}$	Natural mortality rate	^57^
$\mu _1$	$2 \times 10^{-3} \ \text {year}^{-1}$	HBV-related mortality rate	^57^
$\epsilon$	0.16	Reduced transmission rate	^4^
$\gamma _1$	$0.26 \ \text {year}^{-1}$	Progress rate from acute to carrier	^57^
$\gamma _2$	$0.025 \ \text {year}^{-1}$	Progress rate from carrier to immune	^4^
$\gamma _3$	$0.05 \ \text {year}^{-1}$	Vaccination rate for adults	^59^
$q_f$	0.05	Average probability a female adult fails to clear an acute infection and develops to the carrier state	^58^
$q_m$	0.07	Average probability a male adult fails to clear an acute infection and develops to the carrier state	^58^
$\beta$	$3.5 \times 10^{-11} \ \text {year}^{-1}$	Transmission rate for non-sexual transmission	^57^
$\beta _{mf}$	$2.26 \times 10^{-7}$	Transmission probability for males per sex contact with females	^57^
$\beta _{fm}$	$3.06 \times 10^{-3}$	Transmission probability for females per sex contact with males	^57^
$\beta _{ff}$	0.0142	Transmission probability for females per sex contact with females	^57^
$\beta _{mm}$	$9.73 \times 10^{-3}$	Transmission probability for males per sex contact with males	^57^
$c_{mf}$	$21.8 \ \text {year}^{-1}$	Average number of sex contacts of females with males	^57^
$c_{fm}$	$21.8 \ \text {year}^{-1}$	Average number of sex contacts of males with females	^57^
$c_{ff}$	$21.8 \ \text {year}^{-1}$	Average number of sex contacts of females with females	^57^
$c_{mm}$	$65.4 \ \text {year}^{-1}$	Average number of sex contacts of males with males	^57^
$\theta _f$	0.10–0.30	Proportion of female carriers developing chronic disability (10–30%)	Assumed
$\theta _m$	0.15–0.35	Proportion of male carriers developing disability (slightly higher due to HBV severity)	Assumed
$\delta$	0.05 – $0.10 \ \text {year}^{-1}$	Rate of onset of HBV-related disability (10–20 years average latency)	Assumed

## Positivity of solutions

System (2) models the dynamics of hepatitis B infection across multiple gender-based and sexual contact compartments. To guarantee that the state variables $\textbf{S}_f(t), \textbf{I}_f(t), \textbf{C}_f(t), \textbf{S}_m(t), \textbf{I}_m(t), \textbf{C}_m(t), \textbf{R}(t)$ remain non-negative for all $t \ge 0$, it is essential to establish their positivity throughout the time domain.

### Lemma 3.1

*Let* $U \subseteq \mathbb {R}^{n}$* be an open set, and consider a solution* $\varvec{x} = \left( x_{1}, \dots , x_{n} \right) \in C^{1}((0, T); U) \cap C([0, T); U)$* to the ordinary differential equation:*

$${\left\{ \begin{array}{ll} \dot{\varvec{x}}(t) = \varvec{f}(t, \varvec{x}(t)), \\ \varvec{x}(0) = \varvec{x}_{0}, \end{array}\right. }$$

*where the initial condition* $\varvec{x}_{0} = \left( x_{1,0}, \dots , x_{n,0} \right) \in U$* satisfies* $x_{i,0}> 0$* for all* $i \in \{1, \dots , n\}$*, and* $\varvec{f} = \left( f_{1}, \dots , f_{n} \right) : (0, T) \times U \rightarrow \mathbb {R}^{n}$*. Suppose that whenever* $y_{i} = 0$* and all other* $y_{k}> 0$* for* $k \ne i$*, it holds that* $f_{i}(t, \varvec{y})> 0$* for* $t \in (0, T)$*. Then, the solution* $\varvec{x}$ *remains positive for all* $t \in [0, T)$*, meaning *$x_{i}(t)> 0$* for each *$i \in \{1, \dots , n\}$* and* $t \in [0, T)$^60^.

### Theorem 3.1

*The solutions* $\textbf{S}_f(t), \textbf{I}_f(t), \textbf{C}_f(t), \textbf{S}_m(t), \textbf{I}_m(t), \textbf{C}_m(t), \textbf{R}(t)$* of system* (2)* exist for all time and are always positive, smooth, and unique for all* $t> 0$*, subject to the initial conditions*

$$\textbf{S}_f(0), \textbf{I}_f(0), \textbf{C}_f(0), \textbf{S}_m(0), \textbf{I}_m(0), \textbf{C}_m(0), \textbf{R}(0)> 0.$$

### *Proof*

Consider the state vector and its initial values:

$$\varvec{x} = \begin{pmatrix} \textbf{S}_f \\ \textbf{I}_f \\ \textbf{C}_f \\ \textbf{S}_m \\ \textbf{I}_m \\ \textbf{C}_m \\ \textbf{R}\end{pmatrix}, \quad \varvec{x}_0 = \begin{pmatrix} \textbf{S}_f(0) \\ \textbf{I}_f(0) \\ \textbf{C}_f(0) \\ \textbf{S}_m(0) \\ \textbf{I}_m(0) \\ \textbf{C}_m(0) \\ \textbf{R}(0) \end{pmatrix}.$$

Let $\varvec{f} = (f_1, f_2, \dots , f_7)^T$ denote the right-hand side of system (2), where each $f_i$ is defined by the corresponding ODE. These expressions are smooth, locally Lipschitz, and consist of bilinear or linear combinations of state variables with non-negative coefficients (except for natural mortality or transition outflows). Define the total population as:

$$\textbf{N}(t) = \textbf{S}_f + \textbf{I}_f + \textbf{C}_f + \textbf{S}_m + \textbf{I}_m + \textbf{C}_m + \textbf{R}.$$

Since recruitment terms $A_f, A_m$ are constant and death terms are proportional to compartments, $\textbf{N}(t)$ is bounded above, implying that solutions stay within a closed ball $\overline{B}_N \subset U$. By the Picard–Lindelöf theorem^61^, there exists a unique local solution $\varvec{x}(t) \in C^1((0, T); U) \cap C([0, T); U)$. To prove positivity, apply Lemma 3.1. Each $f_i$ remains strictly positive when $x_i = 0$ and the other $x_j> 0$. For instance:

- When $\textbf{S}_f = 0$, we have $\frac{d \textbf{S}_f}{dt} = A_f> 0$;
- When $\textbf{I}_f = 0$, infection and recruitment terms are positive;
- The same holds for $\textbf{C}_f, \textbf{S}_m, \textbf{I}_m, \textbf{C}_m, \textbf{R}$ due to the structure of the ODEs.

Hence, $\varvec{x}(t) \in \mathbb {R}_+^7$ for all $t \ge 0$. By Corollary 17.4 in^62^, the local solution extends globally.

Finally, since $\varvec{f}$ is infinitely differentiable, the solution satisfies the integral equation:

$$\varvec{x}(t) = \varvec{x}_0 + \int _0^t \varvec{f}(\varvec{x}(s)) \, ds.$$

A bootstrap argument implies that $\varvec{x}(t) \in C^\infty$, proving that the solution is smooth, positive, and global. $\square$

## Invariant region and boundedness

We examine system (2) within the biologically relevant feasible region $\mathcal {D}_H$.

### Theorem 4.1

*The region*

$$\mathcal {D}_H = \left\{ (\textbf{S}_f, \textbf{I}_f, \textbf{C}_f, \textbf{S}_m, \textbf{I}_m, \textbf{C}_m, \textbf{R}) \in \mathbb {R}_{+}^{7} : \mathcal {N}(t) \le \frac{A_f + A_m}{\mu _0} \right\}$$

*is positively invariant and attracting for system* (2)* for all *$t \ge 0$*, where* $\mathcal {N}(t) = \textbf{S}_f + \textbf{I}_f + \textbf{C}_f + \textbf{S}_m + \textbf{I}_m + \textbf{C}_m + \textbf{R}$.

### *Proof*

Define the total population size:

$$\mathcal {N}(t) = \textbf{S}_f + \textbf{I}_f + \textbf{C}_f + \textbf{S}_m + \textbf{I}_m + \textbf{C}_m + \textbf{R}.$$

Differentiating with respect to time and summing all equations from system (2), we obtain:

$$\frac{d\mathcal {N}}{dt} = \frac{d\textbf{S}_f}{dt} + \frac{d\textbf{I}_f}{dt} + \frac{d\textbf{C}_f}{dt} + \frac{d\textbf{S}_m}{dt} + \frac{d\textbf{I}_m}{dt} + \frac{d\textbf{C}_m}{dt} + \frac{d\textbf{R}}{dt}.$$

Substituting from system (2), and observing that all terms involving transmission, recovery, and progression cancel internally, we get:

$$\frac{d\mathcal {N}}{dt} = A_f + A_m - \mu _0 \left( \textbf{S}_f + \textbf{I}_f + \textbf{C}_f + \textbf{S}_m + \textbf{I}_m + \textbf{C}_m + \textbf{R}\right) - \mu _1 (\textbf{C}_f + \textbf{C}_m) - \delta (\theta _f \textbf{C}_f + \theta _m \textbf{C}_m).$$

Dropping the non-negative mortality and disability terms (for a bound), we get:

$$\frac{d\mathcal {N}}{dt} \le A_f + A_m - \mu _0 \mathcal {N}.$$

Now apply an integrating factor $I_f = e^{\mu _0 t}$, giving:

$$\frac{d}{dt} \left[ \mathcal {N}(t) e^{\mu _0 t} \right] \le (A_f + A_m) e^{\mu _0 t}.$$

Integrating from $0$ to $t$, we obtain:

$$\mathcal {N}(t) e^{\mu _0 t} - \mathcal {N}(0) \le \frac{A_f + A_m}{\mu _0} \left( e^{\mu _0 t} - 1 \right) ,$$

which simplifies to:

$$\mathcal {N}(t) \le \frac{A_f + A_m}{\mu _0} + \left[ \mathcal {N}(0) - \frac{A_f + A_m}{\mu _0} \right] e^{-\mu _0 t}.$$

Therefore, if $\mathcal {N}(0) \le \frac{A_f + A_m}{\mu _0}$, then $\mathcal {N}(t) \le \frac{A_f + A_m}{\mu _0}$ for all $t \ge 0$, showing positive invariance. If $\mathcal {N}(0)> \frac{A_f + A_m}{\mu _0}$, then $\mathcal {N}(t)$ monotonically decreases and asymptotically approaches this upper bound. Hence, the region $\mathcal {D}_H$ is both positively invariant and attracting, ensuring the biological and mathematical well-posedness of system (2). $\square$

## Basic reproduction number

The model (2) has a disease-free equilibrium given by:

3 $$\begin{aligned} E_0=\left( \textbf{S}_f^0, 0, 0, \textbf{S}_m^0, 0, 0, \textbf{R}^0\right) , \end{aligned}$$

where:

$$\textbf{S}_f^0 = \frac{A_f}{\mu _0 + \gamma _3}, \quad \textbf{S}_m^0 = \frac{A_m}{\mu _0 + \gamma _3}, \quad R^0 = \frac{\gamma _3\left( \textbf{S}_f^0 + \textbf{S}_m^0\right) }{\mu _0} = \frac{\gamma _3\left( A_f + A_m\right) }{\mu _0\left( \mu _0 + \gamma _3\right) }.$$

We calculate the basic reproduction number $R_0$ using the methods in^63,64^. Let:

$$X = \begin{bmatrix} \textbf{I}_f \\ \textbf{I}_m \\ \textbf{C}_f \\ \textbf{C}_m \\ \textbf{S}_f \\ \textbf{S}_m \\ \textbf{R}\end{bmatrix}, \quad \text {and rewrite the system as:} \quad X = \mathscr {F} - \mathscr {V},$$

where:

$$\begin{aligned} & \mathscr {F} = \begin{bmatrix} \lambda \textbf{S}_f + a \lambda _{mf} \textbf{S}_f + (1-a) \lambda _{ff} \textbf{S}_f \\ \lambda \textbf{S}_m + b \lambda _{fm} \textbf{S}_m + (1-b) \lambda _{mm} \textbf{S}_m \\ 0 \\ 0 \\ 0 \\ 0 \\ 0 \end{bmatrix}, \\ & \quad \mathscr {V} = \begin{bmatrix} (\mu _0 + \gamma _1) \textbf{I}_f \\ (\mu _0 + \gamma _1) \textbf{I}_m \\ (\mu _0 + \mu _1 + \gamma _2+ \theta _f \delta ) \textbf{C}_f - q_f \gamma _1 \textbf{I}_f \\ (\mu _0 + \mu _1 + \gamma _2+ \theta _m \delta ) \textbf{C}_m - q_m \gamma _1 \textbf{I}_m \\ -A_f + (\lambda + \mu _0 + \gamma _3) \textbf{S}_f + a \lambda _{mf} \textbf{S}_f + (1-a) \lambda _{ff} \textbf{S}_f \\ -A_m + (\lambda + \mu _0 + \gamma _3) \textbf{S}_m + b \lambda _{fm} \textbf{S}_m + (1-b) \lambda _{mm} \textbf{S}_m \\ -\gamma _3(\textbf{S}_f + \textbf{S}_m) - (1-q_f) \gamma _1 \textbf{I}_f - (1-q_m) \gamma _1 \textbf{I}_m - \gamma _2(\textbf{C}_f + \textbf{C}_m) + \mu _0 \textbf{R}\end{bmatrix}. \end{aligned}$$

The disease-free equilibrium is:

$$\bar{E}_0 = \left( 0, 0, 0, 0, \textbf{S}_f^0, \textbf{S}_m^0, \textbf{R}^0\right) .$$

The Jacobian matrix at $\bar{E}_0$ is given by:

$$D\mathscr {F}\left( \bar{E}_0\right) - D\mathscr {V}\left( \bar{E}_0\right) = \begin{bmatrix} F - V & 0 \\ -H & J \end{bmatrix},$$

where $H$ and $J$ are $3 \times 3$ matrices, and:

$$F = \begin{bmatrix} F_1 & F_2 \\ 0 & 0 \end{bmatrix}, \quad F_1 = \begin{bmatrix} A & B \\ C & D \end{bmatrix}, \quad F_2 = \epsilon F_1.$$

The matrix $V$ is given by:

$$V = \begin{bmatrix} D_1 & 0 \\ -D_2 & V_2 \end{bmatrix},$$

where:

$$D_1 = (\mu _0 + \gamma _1) I_{2 \times 2}, \quad D_2 = \begin{bmatrix} q_f \gamma _1 & 0 \\ 0 & q_m \gamma _1 \end{bmatrix}, \quad V_2 = (\mu _0 + \mu _1 + \gamma _2+ \theta _f \delta ) I_{2 \times 2}.$$

Additionally:

$$A = \beta \textbf{S}_f^0 + \frac{2(1-a) \beta _{ff} c_{ff} \textbf{S}_f^0}{\textbf{N}^0}, \quad B = \beta \textbf{S}_f^0 + \frac{2a \beta _{mf} c_{mf} \textbf{S}_f^0}{\textbf{N}^0},$$

$$C = \beta \textbf{S}_m^0 + \frac{2b \beta _{fm} c_{fm} \textbf{S}_m^0}{\textbf{N}^0}, \quad D = \beta \textbf{S}_m^0 + \frac{2(1-b) \beta _{mm} c_{mm} \textbf{S}_m^0}{\textbf{N}^0}.$$

Following^63,64^, the basic reproduction number $R_0$ is the spectral radius of the matrix $FV^{-1}$, denoted as $\rho (FV^{-1})$. After calculation:

$$R_0 = \frac{r_2 + \sqrt{r_2^2 - 4r_1 r_3}}{2r_1},$$

where:

$$r_1 = (\gamma _1 + \mu _0)^2 (\mu _0 + \mu _1 + \gamma _2+ \theta _f \delta )^2 (\mu _0 + \gamma _3)^2,$$

$$r_2 = (\gamma _1 + \mu _0)(\mu _0 + \mu _1 + \gamma _2+ \theta _f \delta )(\mu _0 + \gamma _3) \left[ A_f\left( \beta + \frac{2(1-a) \beta _{ff} c_{ff}}{N^0}\right) (\mu _0 + \mu _1 + \gamma _2 + \theta _f \delta + \epsilon q_f \gamma _1)\right.$$

$$+ A_m\left( \beta + \frac{2(1-b) \beta _{mm} c_{mm}}{N^0}\right) (\mu _0 + \mu _1 + \gamma _2 + \theta _m \delta + \epsilon q_m \gamma _1)\Big ],$$

$$r_3 = A_f A_m(\mu _0 + \mu _1 + \gamma _2 + \theta _f \delta + \epsilon q_f \gamma _1)(\mu _0 + \mu _1 + \gamma _2 + \theta _m \delta + \epsilon q_m \gamma _1) \left[ \left( \beta + \frac{2(1-a) \beta _{ff} c_{ff}}{N^0}\right) \left( \beta + \frac{2(1-b) \beta _{mm} c_{mm}}{N^0}\right) \right.$$

$$- \left( \beta + \frac{2a \beta _{mf} c_{mf}}{N^0}\right) \left( \beta + \frac{2b \beta _{fm} c_{fm}}{N^0}\right) \Big ],$$

and:

$$N^0 = S_f^0 + S_m^0 + R^0 = \frac{A_f + A_m}{\mu _0}.$$

4 $$\begin{aligned} R_0 = \frac{ \left\{ \begin{aligned}&(\gamma _1 + \mu _0)(\mu _0 + \mu _1 + \gamma _2+ \theta _f \delta )(\mu _0 + \gamma _3) \Bigg [ A_f \left( \beta + \frac{2(1-a)\beta _{ff}c_{ff}}{N^0}\right) (\mu _0 + \mu _1 + \gamma _2 + \theta _f \delta + \epsilon q_f \gamma _1) \\&\quad + A_m \left( \beta + \frac{2(1-b)\beta _{mm}c_{mm}}{N^0}\right) (\mu _0 + \mu _1 + \gamma _2+ \theta _m \delta + \epsilon q_m \gamma _1) \Bigg ] \\&+ \Bigg ( \Bigg [ (\gamma _1 + \mu _0)(\mu _0 + \mu _1 + \gamma _2+ \theta _f \delta )(\mu _0 + \gamma _3) \Big ( A_f \left( \beta + \frac{2(1-a)\beta _{ff}c_{ff}}{N^0}\right) (\mu _0 + \mu _1 + \gamma _2 + \theta _f \delta + \epsilon q_f \gamma _1) \\&\quad + A_m \left( \beta + \frac{2(1-b)\beta _{mm}c_{mm}}{N^0}\right) (\mu _0 + \mu _1 + \gamma _2 + \theta _m \delta + \epsilon q_m \gamma _1) \Big ) \Bigg ]^2 - 4 (\gamma _1 + \mu _0)^2 (\mu _0 + \mu _1 + \gamma _2+ \theta _f \delta )^2\\&\quad (\mu _0 + \gamma _3)^2 \times A_f A_m (\mu _0 + \mu _1 + \gamma _2 + \theta _f \delta + \epsilon q_f \gamma _1)(\mu _0 + \mu _1 + \gamma _2+ \theta _m \delta + \epsilon q_m \gamma _1) \\&\quad \times \Big [ \left( \beta + \frac{2(1-a)\beta _{ff}c_{ff}}{N^0}\right) \left( \beta + \frac{2(1-b)\beta _{mm}c_{mm}}{N^0}\right) - \left( \beta + \frac{2a\beta _{mf}c_{mf}}{N^0}\right) \left( \beta + \frac{2b\beta _{fm}c_{fm}}{N^0}\right) \Big ] \Bigg )^{\frac{1}{2}} \end{aligned} \right\} }{ 2 (\gamma _1 + \mu _0)^2 (\mu _0 + \mu _1 + \gamma _2)^2 (\mu _0 + \gamma _3)^2 }. \end{aligned}$$

## Sensitivity analysis

Sensitivity analysis is a key tool used in infectious disease modeling to identify which parameters most significantly affect disease transmission and control. In particular, forward sensitivity analysis helps determine how small changes in parameters influence key epidemiological outcomes such as the basic reproduction number $R_0$. Although the computation can become challenging for complex biological models, this technique remains essential. Sensitivity analysis of $R_0$ has been widely employed by ecologists and epidemiologists to guide effective intervention strategies.

### Definition 6.1

The normalized forward sensitivity index of $R_0$ that depends differentiably on a parameter $\bar{\Omega }$ is defined as

$$\begin{aligned} S_\Omega =\frac{\bar{\Omega }}{R_0}\frac{\partial R_0}{\partial \bar{\Omega }}. \end{aligned}$$

Three methods are commonly used to calculate sensitivity indices: (i) by direct differentiation, (ii) by a Latin hypercube sampling method, and (iii) by linearizing the system (2) and then solving the obtained set of linear algebraic equations. We will apply the direct differentiation method as it provides analytical expressions for the indices. The indices not only show us the influence of various aspects associated with the spread of infectious diseases but also provide important information regarding the comparative change between $R_0$ and different parameters. Consequently, it helps in developing effective control strategies.

Table 2 shows that the parameters $A_f$, $A_m$, $\beta$, $q_f$, $q_m$, $\epsilon$, $\beta _{ff}$, $\beta _{mm}$, $c_{ff}$, $c_{mm}$, $\theta _m$, and $\delta$ positively influence the reproduction number $R_0$. This implies that a 10% increase or decrease in these parameters will proportionally increase or decrease $R_0$ by 3.6726%, 6.3274%, 2.6193%, 0.21231%, 0.50053%, 0.71284%, 2.3779%, 5.0025%, 2.3779%, 5.0025%, 2.7142%, and 0.22253%, respectively. On the other hand, the indices for parameters $a$, $b$, $\gamma _1$, $\gamma _2$, $\gamma _3$, $\mu _0$, $\mu _1$, $\beta _{mf}$, $\beta _{fm}$, $c_{mf}$, $c_{fm}$, and $\theta _f$ indicate that increasing their values by 10% will reduce $R_0$ by 7.1338%, 15.007%, 13.793%, 4.1326%, 13.085%, 3.3313%, 0.33061%, $3.3145 \times 10^{-8}\%$, $2.0515 \times 10^{-4}\%$, $3.3145 \times 10^{-8}\%$, $2.0515 \times 10^{-4}\%$, and 2.4917%, respectively. These results underscore the importance of prioritizing interventions that target the most sensitive parameters, such as $b$, $\gamma _1$, $\gamma _3$, and now $\theta _f$ and $\theta _m$, to effectively manage $R_0$ and control HBV transmission.

**Table 2:** Sensitivity indices of the reproduction number $R_0$ against mentioned parameters, including disability dynamics.

Parameter	$S_{\text {Index}}$	Value	Parameter	$S_{\text {Index}}$	Value
*a*	$S_a$	−0.71338	*b*	$S_b$	−1.5007
$A_f$	$S_{Af}$	0.36726	$A_m$	$S_{Am}$	0.63274
$\beta$	$S_{\beta }$	0.26193	$\gamma _1$	$S_{\gamma _1}$	−1.3793
$\gamma _2$	$S_{\gamma _2}$	−0.41326	$\gamma _3$	$S_{\gamma _3}$	−1.3085
$\mu _0$	$S_{\mu _0}$	−0.33313	$\mu _1$	$S_{\mu _1}$	−0.033061
$q_f$	$S_{qf}$	0.021231	$q_m$	$S_{qm}$	0.050053
$\epsilon$	$S_{\epsilon }$	0.071284	$\beta _{ff}$	$S_{\beta _{ff}}$	0.23779
$\beta _{mm}$	$S_{\beta _{mm}}$	0.50025	$\beta _{mf}$	$S_{\beta _{mf}}$	3.3145e−09
$\beta _{fm}$	$S_{\beta _{fm}}$	2.0515e−05	$c_{mf}$	$S_{cmf}$	3.3145e−09
$c_{fm}$	$S_{cfm}$	2.0515e−05	$c_{ff}$	$S_{cff}$	0.23779
$c_{mm}$	$S_{cmm}$	0.50025	$\theta _f$	$S_{\theta _f}$	−0.24917
$\theta _m$	$S_{\theta _m}$	0.27142	$\delta$	$S_{\delta }$	0.022253

**Fig. 1:**
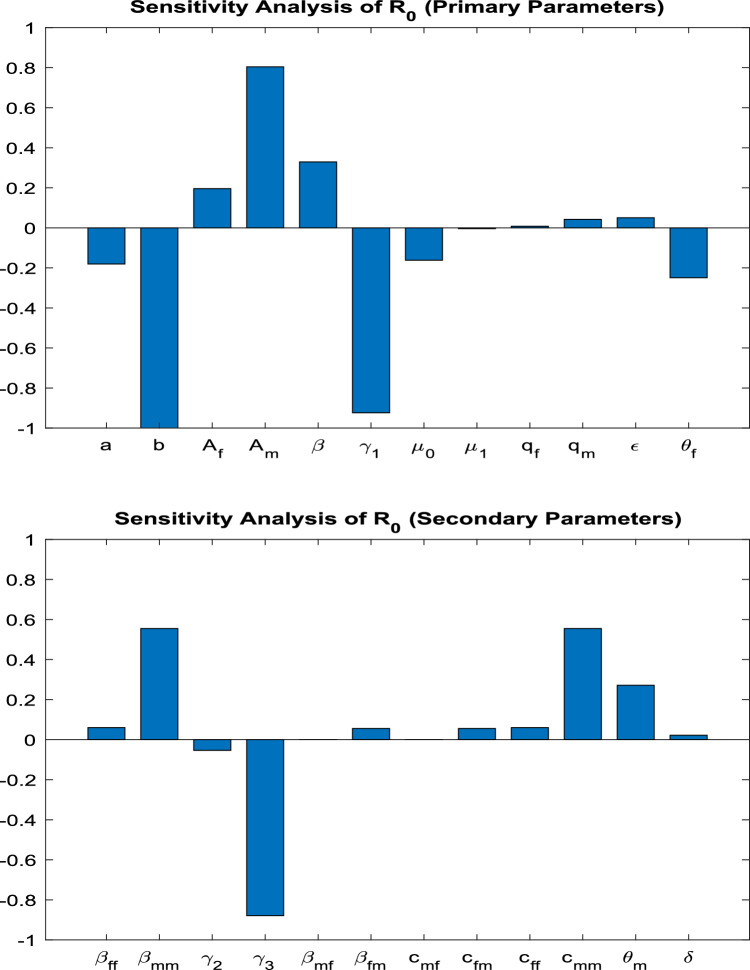
Sensitivity analysis of the basic reproduction number ($R_0$) with respect to model parameters. Positive and negative bars indicate the parameters that increase or decrease $R_0$, respectively, highlighting their relative influence on disease transmission dynamics.

**Fig. 2:**
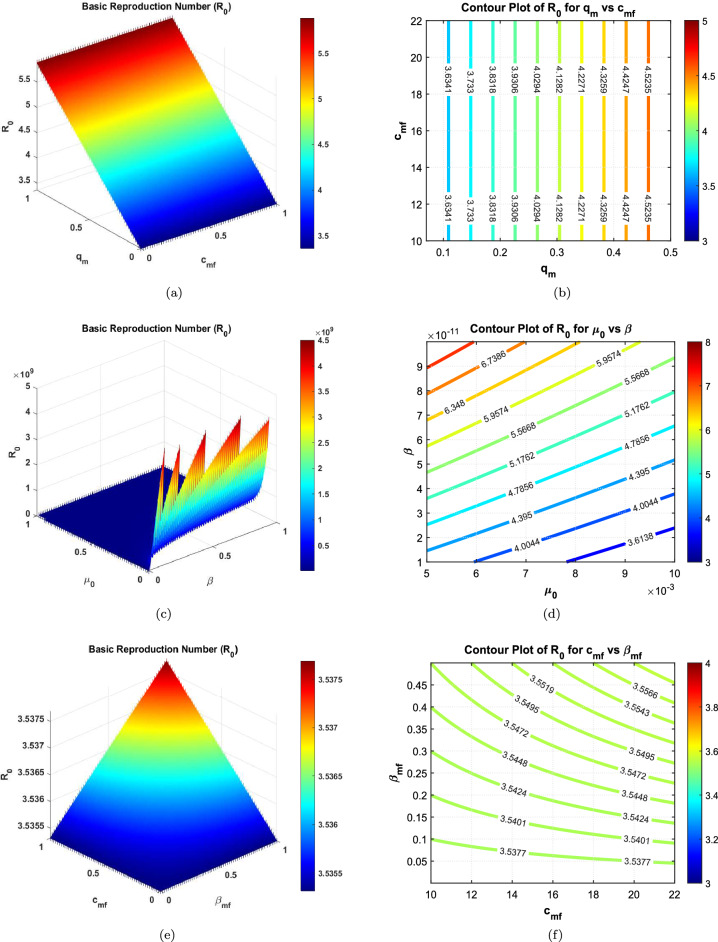
3D surface and contour plots showing the influence of various parameter pairs on $R_0$ in the proposed HBV model. Subfigures (**a**) and (**b**) illustrate the joint impact of the male infection clearance probability $q_m$ and the male-to-female contact rate $c_{mf}$. Subfigures (**c**) and (**d**) analyze how the natural mortality rate $\mu _0$ and non-sexual transmission rate $\beta$ shape $R_0$. Subfigures (**e**) and (**f**) explore the sensitivity of $R_0$ to the interaction between male-to-female contact rate $c_{mf}$ and male-to-female transmission probability $\beta _{mf}$. These visualizations help identify key drivers in transmission dynamics and potential intervention targets.

**Fig. 3:**
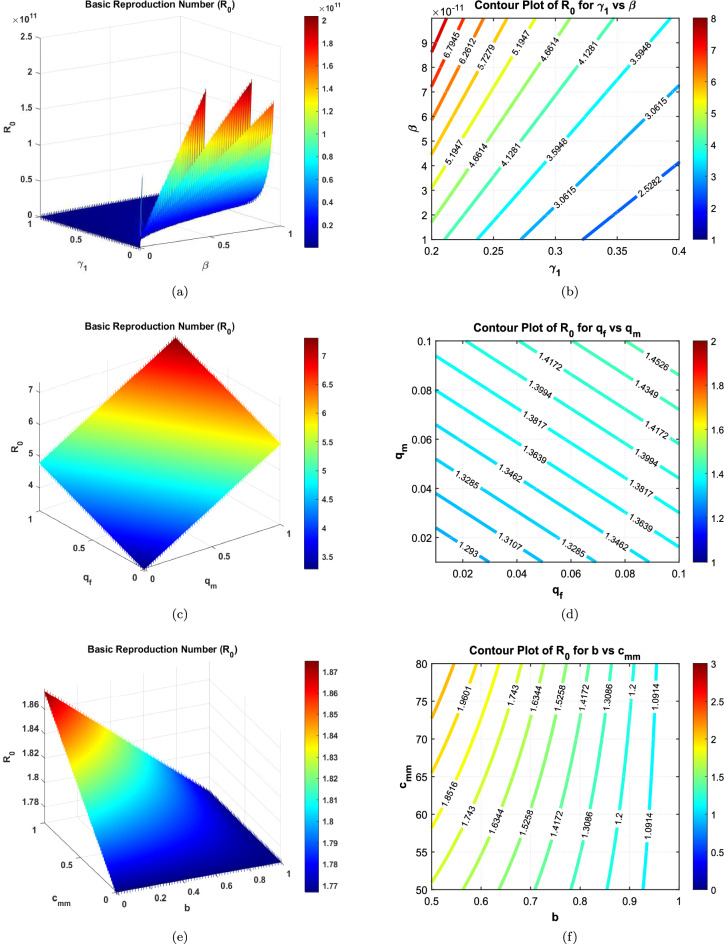
3D surface and contour plots demonstrating the influence of selected parameter pairs on $R_0$ in the HBV model. Subfigures (**a**) and (**b**) illustrate the interaction between the acute-to-carrier progression rate $\gamma _1$ and the non-sexual transmission rate $\beta$. Subfigures (**c**) and (**d**) show the combined effect of the probabilities of failed infection clearance in females and males ($q_f$, $q_m$). Subfigures (**e**) and (**f**) depict the impact of reduced transmission rate $\epsilon$ and the male-to-male contact rate $c_{mm}$. These plots highlight the parameter dependencies and nonlinear sensitivities in the transmission dynamics.

### Analysis and discussion of sensitivity results

The apparent sensitivity analysis performed on the basic reproduction number $R_0$ aids in comprehending the dynamics of the transmission of the HBV in the population under consideration which has been modelled. In this section, the important parameters regulating $R_0$ are detailed and their physical and biological roles are discussed from the context of public health policy-making.

#### Key influencing parameters

The results clearly show that a few parameters increase the $R_0$ considerably. Gender-based recruitment rates ($A_m$ and $A_f$), probabilities of transmission ($\beta _{mf}$, $\beta _{fm}$, $\beta _{mm}$, $\beta _{ff}$), and contact rates ($c_{mf}$, $c_{fm}$, $c_{mm}$, $c_{ff}$) exhibit a strong positive correlation with $R_0$. These parameters are directly related to the sexual activity involved in having may be children of susceptible parents hence increasing the size of a group of infected:

- **Recruitment rates** The sensitivity indices related to $A_m$ and $A_f$ suggest that increasing the flow of susceptible into the population considerably increases $R_0$. This points to the need of dealing with factors that explain the increase in the size of the population like immigration and recruitment some more.
- **Transmission probabilities** The high sensitivity of $R_0$ to $\beta _{mm}$ and $\beta _{ff}$ highlights the significant risk posed by male-to-male and female-to-female sexual transmission pathways. This aligns with epidemiological studies indicating that these pathways often have higher efficiency and transmission dynamics.
- **Contact rates** The parameters $c_{mm}$ and $c_{ff}$ represent the frequency of sexual contacts in specific sub-populations. The high sensitivity index for $c_{mm}$, in particular, suggests the importance of targeted behavioral interventions within male-to-male sexual networks.

#### Mitigating factors

Several parameters in the model exhibit a negative influence on the basic reproduction number ($R_0$), effectively acting as control levers for reducing disease transmission. These include traditional epidemiological interventions such as vaccination rates, disease progression rates, reduced transmission rates, and disability-related parameters.

- **Vaccination (**$\gamma _3$**)** Among all mitigating factors, $\gamma _3$ has the strongest suppressive effect on $R_0$. This emphasizes the critical role of adult vaccination campaigns in interrupting HBV transmission chains and decreasing the pool of susceptible individuals.
- **Disease progression (**$\gamma _1$, $\gamma _2$**)** The progression parameters impact the transition from acute infection to carrier state and from carrier to recovered state. Early diagnosis and clinical interventions that accelerate recovery or limit chronic progression contribute significantly to reducing long-term infectivity.
- **Reduced transmission rate (**$\epsilon$**)** This parameter captures the effectiveness of behavioral and biomedical interventions, such as condom use, education, and antiretroviral therapies, that reduce the probability of virus transmission per contact.
- **Female disability rate (**$\theta _f$**)** A novel addition, $\theta _f$ reflects the proportion of female carriers who develop HBV-related disability. Its negative influence on $R_0$ suggests that disability shortens the functional infectious period, thereby indirectly reducing secondary infections.
- **Male disability rate (**$\theta _m$**)** Interestingly, $\theta _m$ has a slight positive effect on $R_0$. This may point to gender-based differences in disease progression, behavior, or access to healthcare, wherein male carriers may continue to contribute to transmission before experiencing debilitating symptoms.
- **Disability onset speed (**$\delta$**)** The parameter $\delta$ quantifies how quickly disability becomes clinically evident in carriers. Higher values of $\delta$ modestly reduce $R_0$, underscoring the importance of timely detection and support for affected individuals to limit their transmission potential.

From an epidemiological standpoint, the parameters $\theta _f$, $\theta _m$, and $\delta$ jointly capture the complex interplay between biological progression and social factors influencing HBV transmission. A higher value of $\theta _f$ implies that female carriers are more likely to develop disability earlier, shortening their infectious period and thereby reducing $R_0$. Conversely, the mild positive sensitivity of $R_0$ to $\theta _m$ may reflect delayed clinical progression and prolonged infectious activity among males, who often experience slower onset of HBV-related complications and may engage in riskier sexual behavior or face barriers to early medical care. The parameter $\delta$ modulates the overall speed of disability onset across both genders, indicating that rapid progression to advanced disease states diminishes the duration of infectiousness and hence suppresses transmission potential. These findings reinforce the importance of gender-sensitive clinical management and early diagnosis in limiting the long-term burden of HBV.

#### Graphical interpretations

The graphical representations in Figs. 1, 2, and 3 provide a comprehensive view of the parameter interplay:

- **Bar graphs** Figures 1 clearly illustrate the relative sensitivity of $R_0$ to each parameter. Positive bars indicate parameters that increase $R_0$, while negative bars highlight parameters that reduce it. The steep gradients in parameters such as $\gamma _3$ and $c_{mm}$ demonstrate their critical role in transmission dynamics.
- **3D contours** Figures 2 and 3 depict the interactions between pairs of parameters. For instance, the combination of $\gamma _3$ and $\epsilon$ shows a synergistic effect in reducing $R_0$, providing actionable insights for optimizing public health interventions.

#### Physical interpretation and public health implications

From a physical standpoint, the parameters act as forces driving or dampening the transmission dynamics. For example:

- Parameters such as $\beta _{mm}$ and $c_{mm}$ serve as driving forces, accelerating the spread of the virus by increasing the number of effective contacts.
- Conversely, vaccination ($\gamma _3$) and reduced transmission ($\epsilon$) act as dissipative forces, stabilizing the system and driving it toward a disease-free equilibrium.

These findings underline the necessity of prioritizing high-impact interventions, such as scaling up vaccination efforts, promoting safe sex practices, and implementing targeted behavioral interventions for high-risk populations. By addressing the most sensitive parameters, policymakers can achieve significant reductions in $R_0$ and effectively curb the spread of HBV.

## ANN error analysis and training via LM algorithm

To improve computational efficiency in solving the proposed HBV model, we integrate an ANN framework with the LMB optimization algorithm. This section outlines the reference numerical method used for generating training data, the ANN training procedure, and the optimization strategy employed.

### Numerical reference using Runge-Kutta method

The RK4 scheme is adopted to generate high-accuracy numerical solutions of the system of differential equations governing HBV transmission. For an ordinary differential equation of the form

$$\frac{dY}{dt} = F(t, Y),$$

the solution at the next time step is approximated as

$$Y_{n+1} = Y_{n} + \frac{h}{6} (G_{1} + 2G_{2} + 2G_{3} + G_{4}),$$

where

$$G_{1} = F(t_n, Y_n), \quad G_{2} = F\!\left( t_n + \tfrac{h}{2}, Y_n + \tfrac{h}{2} G_{1}\right) ,$$

$$G_{3} = F\!\left( t_n + \tfrac{h}{2}, Y_n + \tfrac{h}{2} G_{2}\right) , \quad G_{4} = F\!\left( t_n + h, Y_n + h G_{3}\right) .$$

Here *h* denotes the step size and *F*(*t*, *Y*) represents the right–hand side of the HBV system. These RK4 outputs act as reference solutions, which are later used to train the ANN.

**Rationale for synthetic data use** The ANN in this study was trained on numerical solutions generated by the differential-equation model rather than on field observations. This design was chosen to evaluate the network’s ability to emulate the mechanistic model dynamics under controlled, noise-free conditions. Training on synthetic data ensures that reference outputs fully reflect model assumptions and parameter interactions, providing a precise benchmark for assessing the ANN’s approximation capability. Accordingly, the ANN functions as a computational surrogate that accelerates model evaluation and sensitivity exploration, rather than as a forecasting tool for real-world HBV outbreaks. Future work will extend this framework to integrate empirical or surveillance data for predictive validation.

### Training strategy and dataset partitioning

The ANN is trained to approximate the trajectories of each epidemiological compartment by learning from the RK4 solutions. To avoid overfitting and ensure generalization, the dataset is divided into:

- **Training set:** used to update network weights and biases,
- **Validation set:** monitors model generalization and halts training when overfitting is detected,
- **Testing set:** reserved for final evaluation of predictive performance.

The accuracy of the ANN predictions is quantified using the mean squared error (MSE):

$$MSE = \frac{1}{n} \sum _{i=1}^{n} \big (y_i - \hat{y}_i \big )^2,$$

where $y_i$ are the RK4 reference values and $\hat{y}_i = O(\theta )$ denotes the ANN output corresponding to network parameters $\theta$.

### Levenberg–Marquardt optimization

The LMB algorithm is employed to minimize the prediction error of the ANN. The error function is expressed as

$$E(\theta ) = \tfrac{1}{2} \sum _{i=1}^{n} (y_i - \hat{y}_i)^2.$$

The parameter update rule is given by

$$\theta ^{new} = \theta ^{old} - (J^T J + \lambda I)^{-1} J^T e,$$

where *J* is the Jacobian matrix of partial derivatives of the error with respect to $\theta$, $e = y - \hat{y}$ is the error vector, $\lambda$ is a damping factor regulating the step size, and *I* is the identity matrix. This hybrid approach combines the speed of the Gauss–Newton method with the stability of gradient descent, making it well suited for nonlinear problems such as Lassa HBV dynamics.

**Scientific role of the ANN** In this framework, the ANN does not function as a forecasting tool for real epidemics but as a computational surrogate that emulates the numerical behavior of the HBV transmission model. By replacing repeated numerical integrations with rapid neural approximations, the ANN enables efficient exploration of parameter sensitivities and transmission pathways. This approach provides indirect biological insights by revealing how model dynamics respond to variations in key epidemiological and disability-related parameters without relying on empirical data.

**Model validation and scope** The present work focuses on validating the ANN’s ability to emulate the mechanistic HBV model under controlled, noise-free conditions using synthetic data generated via the RK4 solver. Although the framework was not trained on real epidemiological or cohort data, this design provides a precise computational benchmark for assessing numerical fidelity and parameter sensitivity. Integration with empirical datasets is identified as an important direction for future work, which will allow direct performance comparison between the surrogate ANN model and traditional mechanistic or statistical approaches in real-world settings.

### Error distribution via histograms

To evaluate how well the ANN reproduces the compartmental trajectories of the HBV model, error histograms are constructed. These plots highlight how prediction errors are distributed across the training, validation, and testing sets. For each time point *t*, the error is defined as

$$e(t) = y(t) - \hat{y}(t),$$

where *y*(*t*) denotes the RK4 reference solution and $\hat{y}(t) = O(\theta , t)$ is the ANN output generated with parameters $\theta$. A narrow, symmetric distribution of *e*(*t*) values around zero indicates accurate approximation and good generalization.

### Regression analysis and correlation assessment

The consistency between ANN predictions and RK4 reference values is further assessed through regression analysis. The correlation coefficient (*R*) is computed as

$$R = \frac{\sum _{i=1}^{n} \big (y_i - \bar{y}\big )\big (\hat{y}_i - \bar{\hat{y}}\big )}{\sqrt{\sum _{i=1}^{n} (y_i - \bar{y})^2}\; \sqrt{\sum _{i=1}^{n} (\hat{y}_i - \bar{\hat{y}})^2}},$$

where $y_i$ represents the RK4 reference values, $\hat{y}_i$ are the ANN predictions, and $\bar{y}$, $\bar{\hat{y}}$ denote their respective means. An *R* value close to 1 demonstrates that the ANN is able to closely track the underlying epidemic trajectories.

### Performance metrics and error evolution

Training performance is monitored through the evolution of the mean squared error (MSE) over epochs. Tracking the decline of MSE provides insight into convergence behavior and the network’s ability to learn the nonlinear dynamics of HBV transmission. The instantaneous absolute error at time *t* is given by

$$e(t) = \big | y(t) - \hat{y}(t) \big |,$$

which can be examined to identify periods where the ANN performs less accurately, such as during epidemic peaks or rapid transitions in the system.

## Proposed methodology: ANN-LMB approach

**Fig. 4:**
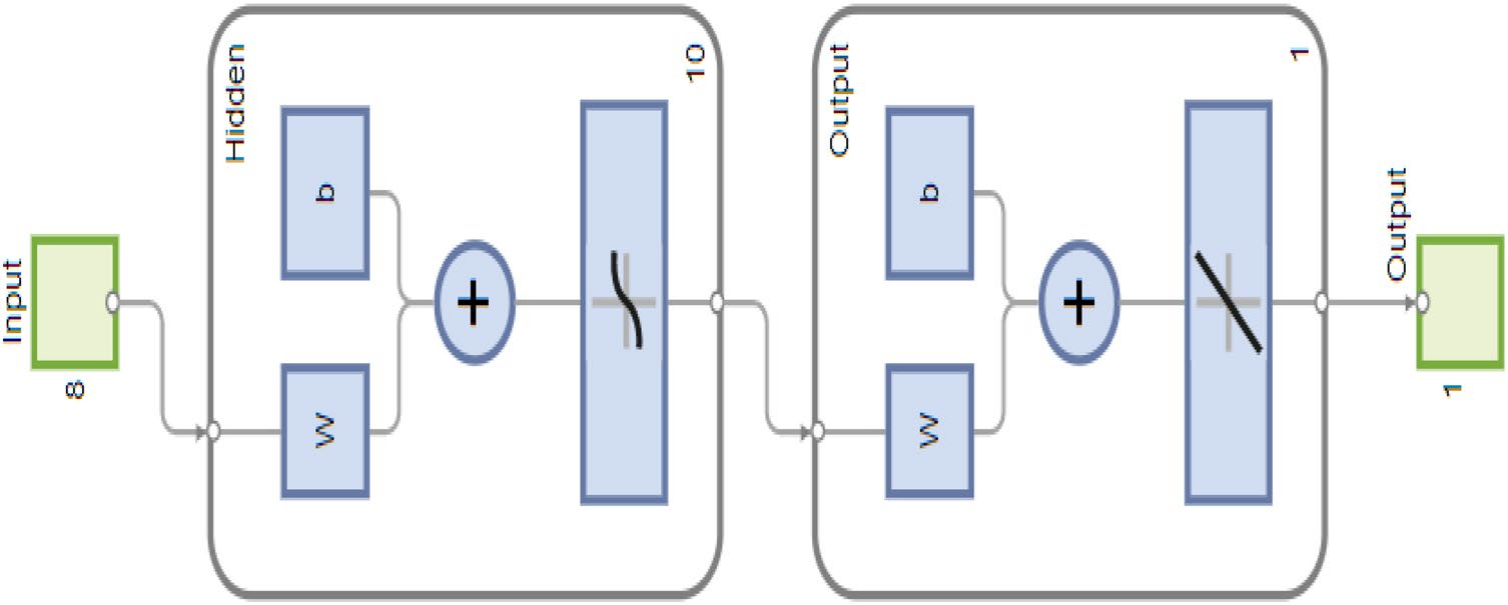
Structural layout of the ANN-LMB model, comprising input, hidden (10 neurons), and output layers.

To complement the analytical results and accelerate simulations of the HBV model, we employ an ANN trained with the LMB algorithm. The ANN–LMB framework is designed to approximate the nonlinear dynamics of the compartmental system while reducing computational cost compared to repeated numerical integration. The methodology consists of two main stages: (i) generation of training data from the RK4 solutions of the HBV equations, and (ii) supervised learning of the ANN using the LMB optimization routine. All computations were performed in MATLAB (Neural Fitting tool). The dataset was randomly partitioned into three subsets: 85% for training, 10% for validation, and 5% for testing. This partitioning strategy ensures that the network is exposed to diverse epidemic scenarios during training, while validation and testing safeguard against overfitting and provide an unbiased measure of predictive accuracy. The proposed ANN-LM model consists of an input layer, a single hidden layer with 10 neurons, and an output layer. This simple but flexible structure was sufficient to capture the nonlinear relationships among model compartments. The number of hidden neurons (10) was selected after several trial configurations (5–15 neurons), with this setup providing the most stable convergence and minimal mean-squared error, while maintaining computational efficiency. Figure 4 illustrates the architecture of the network. The interconnected layers collectively approximate the progression of HBV across different compartments, offering insight into both epidemic growth and control interventions.

### Artificial neuron structure

The elementary computational unit of the ANN is the artificial neuron, originally introduced by McCulloch and Pitts^65^. A neuron receives multiple inputs, applies a weighted sum, and passes the result through an activation function to generate its output. Mathematically, this is expressed as

5 $$\begin{aligned} Y = \varphi \left( \sum _{i=1}^{n} w_i x_i - \theta \right) , \end{aligned}$$

where $\varphi$ is the activation function, $\theta$ is the threshold, and $w_i$, $x_i$ denote the weight and the input of the *i*-th connection. Including a bias term *b* in place of the threshold gives

6 $$\begin{aligned} Y = \varphi \left( \sum _{i=1}^{n} w_i x_i + b \right) . \end{aligned}$$

**Fig. 5:**
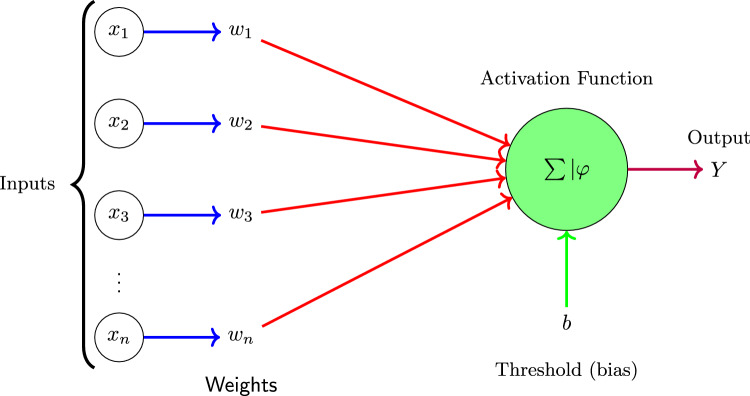
Schematic representation of a single artificial neuron, illustrating how weighted inputs are combined with a bias and passed through an activation function to produce an output^65^.

Both linear and nonlinear activation functions may be employed; in practice, nonlinear choices such as the hyperbolic tangent sigmoid $\varphi (k) = \tanh (k)$ are favored for capturing complex epidemic patterns. Figure 5 shows a schematic diagram of an artificial neuron.

## Simulation results and discussion

**Fig. 6:**
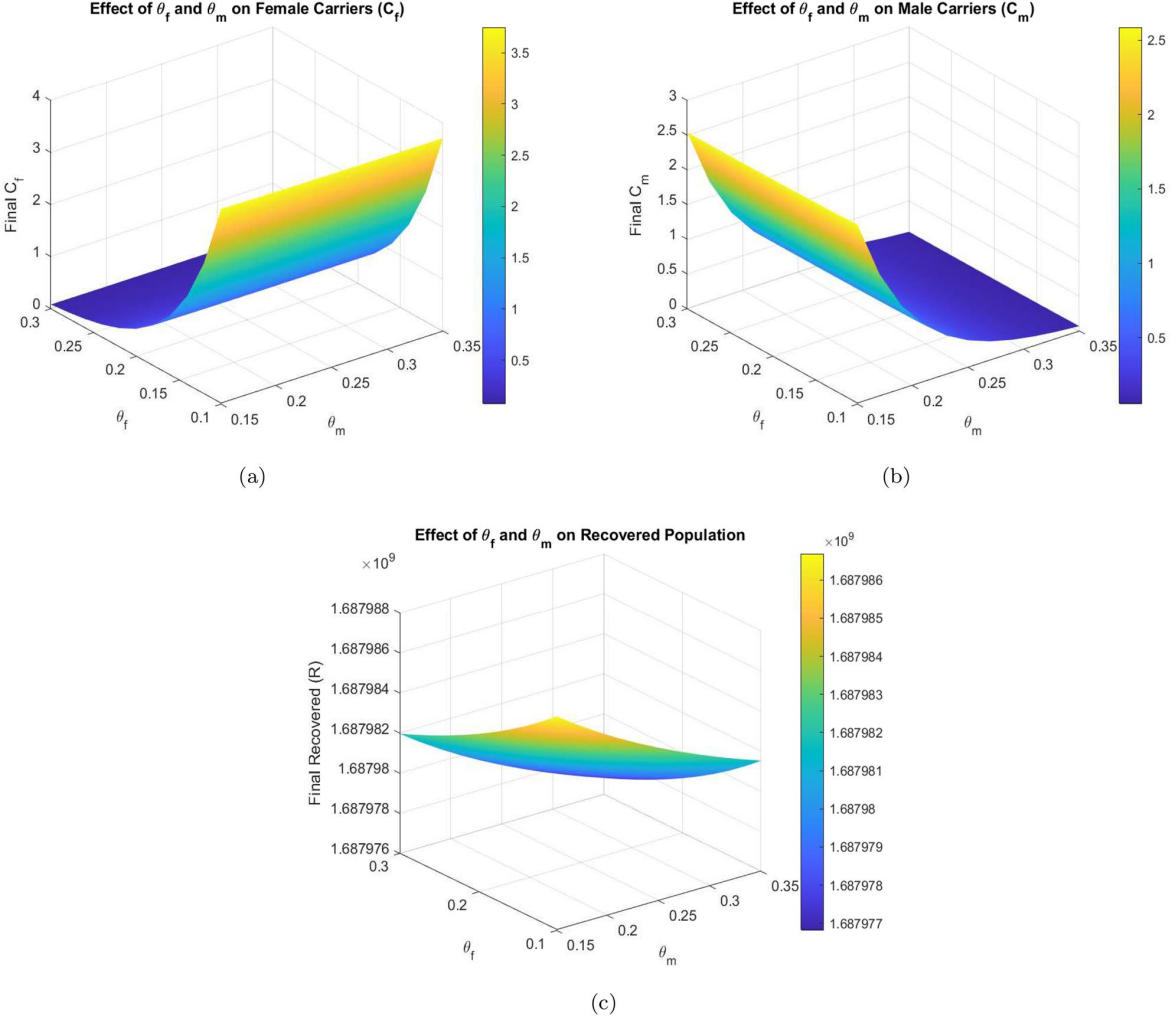
Influence of disability onset probabilities $\theta _f$ and $\theta _m$ on final disease outcomes. Subfigures show the long-term values of (**a**) female carriers $C_f$, (**b**) male carriers $C_m$, and (**c**) the recovered population *R*(*t*). As $\theta _f$ and $\theta _m$ increase, both $C_f$ and $C_m$ decrease due to earlier disability onset, while *R*(*t*) exhibits modest nonlinear variation.

**Fig. 7:**
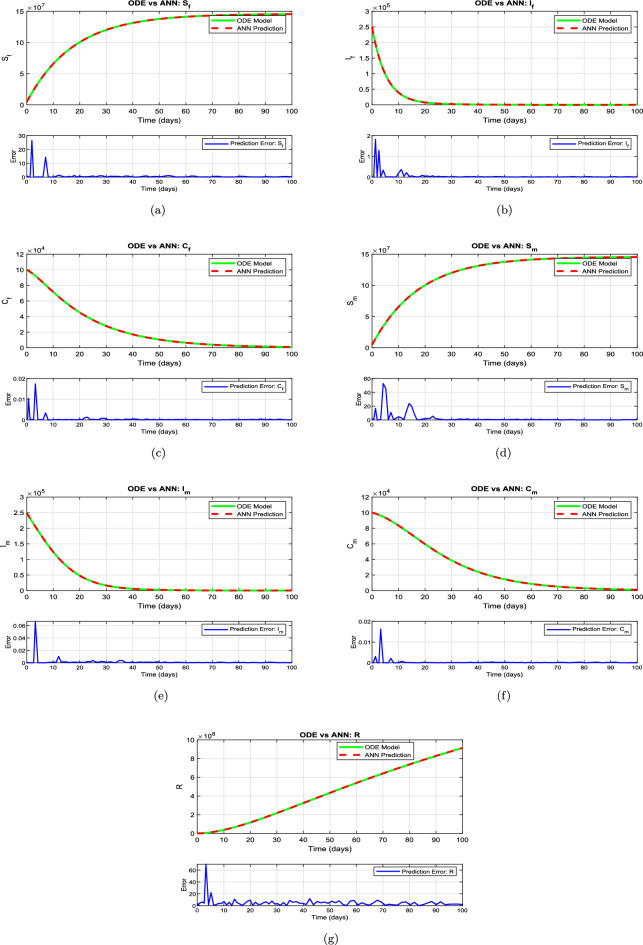
Comparison between the numerical solutions of the proposed HBV model 2 using the Runge–Kutta method and the corresponding predictions generated by the ANN–LMB model.

**Fig. 8:**
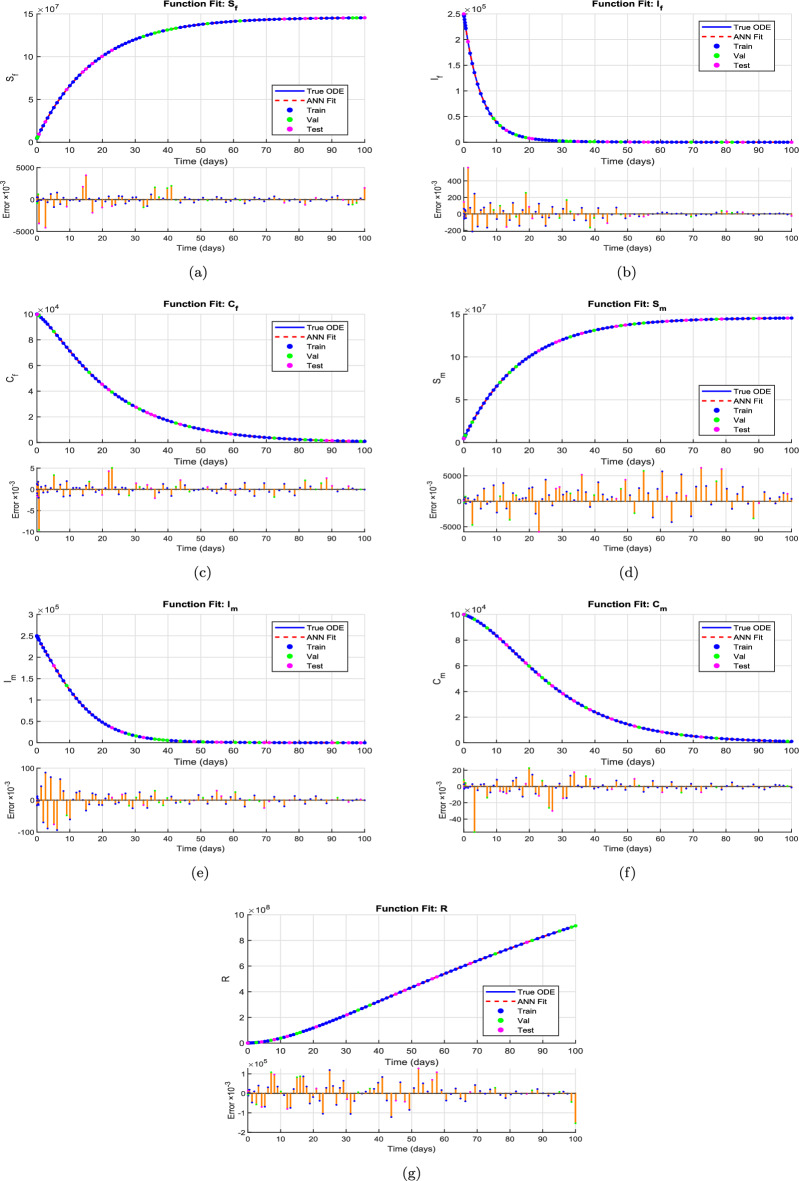
Function fitting results comparing the true ODE-based solutions and the ANN–LMB predictions for each compartment. The plots include outputs from training, validation, and testing subsets. Bar plots indicate corresponding prediction errors across the time horizon.

**Fig. 9:**
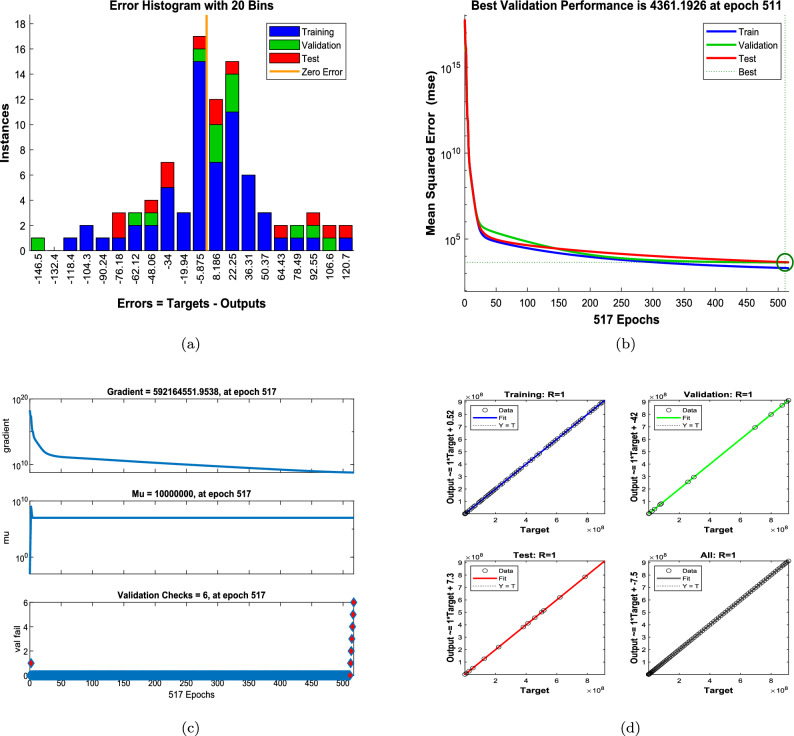
ANN–LMB training diagnostics and performance evaluation. (**a**) Error histogram displaying prediction residuals across training, validation, and test sets. (**b**) Mean squared error (MSE) over training epochs, highlighting the best validation performance at epoch 511. (**c**) Convergence metrics including gradient norm, damping parameter $\mu$, and validation checks. (**d**) Regression plots showing the correlation between target and predicted values for training, validation, test, and combined datasets with $R \approx 1$ in all cases.

**Fig. 10:**
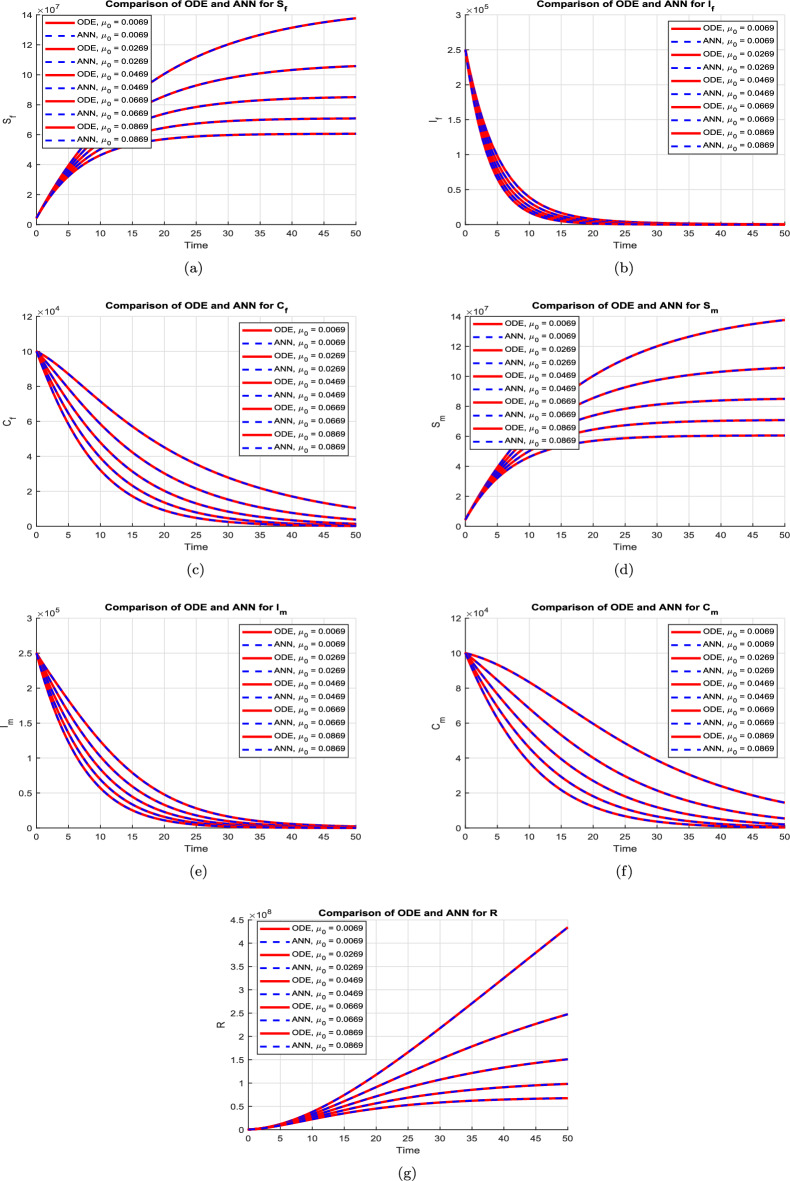
Comparison of numerical solutions (ODE) and ANN predictions for different values of the natural mortality rate $\mu _0$. Each subfigure shows how increasing $\mu _0$ influences the compartmental dynamics, with solid lines representing RK4 solutions and dashed lines denoting ANN outputs.

**Fig. 11:**
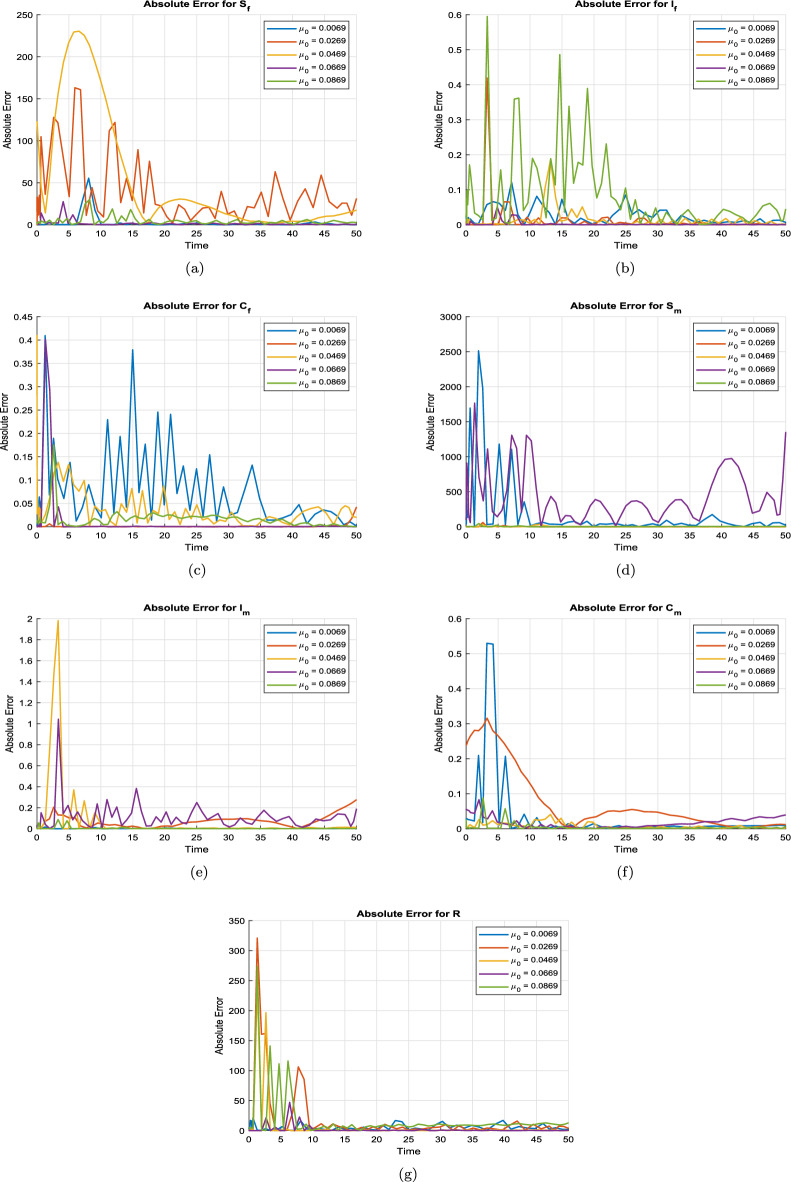
Absolute errors between ANN predictions and ODE solutions for varying natural mortality rates $\mu _0$. The results highlight the influence of $\mu _0$ on prediction accuracy across different compartments.

**Fig. 12:**
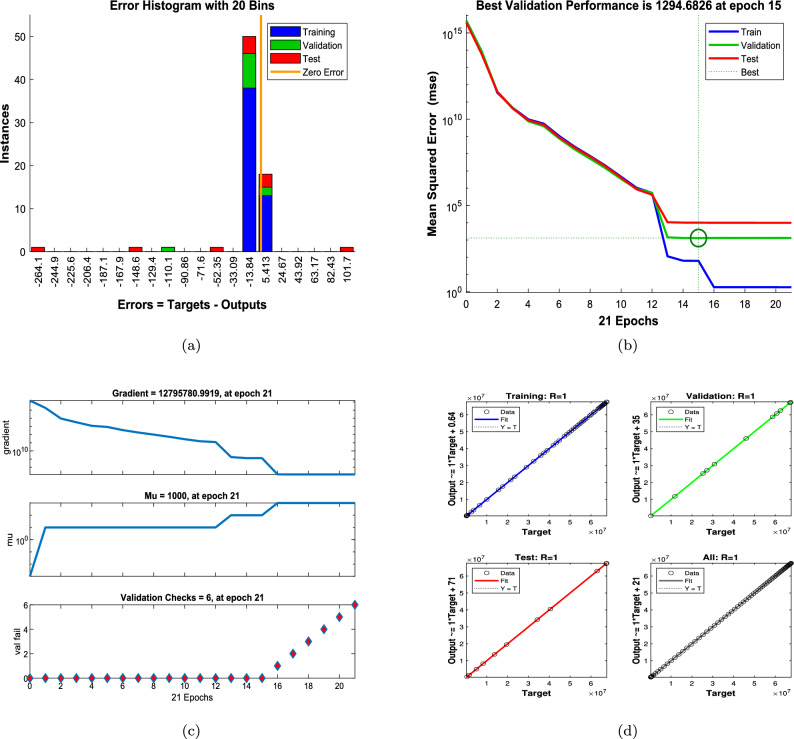
ANN–LMB training diagnostics for the HBV model with varying natural mortality rate $\mu _0$. (**a**) Histogram showing error distribution across training, validation, and test sets. (**b**) MSE performance over 21 training epochs, with best validation performance achieved at epoch 15. (**c**) Training convergence metrics including gradient norm, damping parameter $\mu$, and validation checks. (**d**) Regression plots for training, validation, test, and all datasets, each showing strong correlation ($R \approx 1$) between predicted and target values.

This section presents the computational analysis of the hepatitis B virus transmission model, incorporating the effects of disability progression, sex-specific compartments, and long-term health impact. Model solutions were obtained using the parameter values outlined in Table 1. To evaluate the model’s robustness and predictive capability, simulations were performed using two complementary numerical approaches: the classical RK4 method and an ANN framework trained via the LMB algorithm. Figures 6, 7, 8, and 10 provide comparative visualizations of the RK4 numerical solutions and the corresponding ANN–LMB predictions across various epidemiological compartments, including susceptible, infected, chronic, and recovered populations. The associated absolute error plots are shown in Figs. 9, 11, and 12, confirming the high predictive accuracy of the ANN model. Across all compartments, the ANN approach exhibits excellent agreement with the RK4 benchmark, with absolute errors remaining consistently low throughout the simulation horizon. To assess the epidemiological implications of HBV-induced disability, additional simulations were conducted under varying values of the disability-related parameters $\theta _f$, $\theta _m$, and $\delta$. The results show that increasing the probabilities $\theta _f$ and $\theta _m$ leads to a reduction in the number of functionally healthy carriers, despite the total carrier population remaining constant. This decline in the functional carrier pool reflects a hidden burden of disease not typically captured in standard compartmental models. Moreover, higher values of $\delta$, representing faster progression to disability, further shorten the duration during which carriers remain productive, emphasizing the need for timely medical intervention and preventive care.

We simulate the mathematical model (2) to analyze the impact of specific parameters on the dynamics of HBV transmission. Simulations are conducted over a period of 100 days to observe the system’s long-term behavior and stability. To track the dynamics of infected individuals in both adult female and male populations, we define the following normalized state variables:

$$x_1 = \frac{\textbf{S}_f}{\textbf{N}}, \quad x_2 = \frac{\textbf{I}_f}{\textbf{N}}, \quad x_3 = \frac{\textbf{C}_f}{\textbf{N}}, \quad x_4 = \frac{\textbf{S}_m}{\textbf{N}}, \quad x_5 = \frac{\textbf{I}_m}{\textbf{N}}, \quad x_6 = \frac{\textbf{C}_m}{\textbf{N}}, \quad x_7 = \frac{\textbf{R}}{\textbf{N}}.$$

The initial values of these variables are specified as:

$$x_1(0) = \frac{\textbf{S}_f(0)}{\textbf{N}(0)} = 0.42, \quad x_2(0) = \frac{\textbf{I}_f(0)}{\textbf{N}(0)} = 0.000296771, \quad x_3(0) = \frac{\textbf{C}_f(0)}{\textbf{N}(0)} = 8.09 \times 10^{-3},$$

$$x_4(0) = \frac{\textbf{S}_m(0)}{\textbf{N}(0)} = 0.47, \quad x_5(0) = \frac{\textbf{I}_m(0)}{\textbf{N}(0)} = 0.000575071, \quad x_6(0) = \frac{\textbf{C}_m(0)}{\textbf{N}(0)} = 7.3 \times 10^{-4}.$$

The total initial population is taken as $\textbf{N}(0) = 931,400,000$. These initial conditions, where applicable, are optimized using MATLAB and derived from data provided by the CDC^57^. The numerical values of all parameters used in the simulations are presented in Table 1.

Figure 7 presents a comparative analysis of the numerical solutions obtained from the proposed HBV model (2) using the classical Runge–Kutta method and the predictions generated by the ANN–LMB framework. As illustrated in Fig. 7a, the susceptible female population $\textbf{S}_f(t)$ exhibits an exponential rise that eventually levels off, indicating a reduction in infection pressure or increasing immunity over time. The ANN prediction aligns closely with the ODE solution, with minimal error evident from the error subplot. In subfigure 7b, the infected female class $\textbf{I}_f(t)$ rapidly declines toward zero within the first 20 days, reflecting swift progression out of the acute infection state, either to recovery or chronic infection. The ANN model accurately reproduces this decay, with error remaining consistently low beyond the early transient phase. Figure 7c shows the dynamics of the chronically infected female population $\textbf{C}_f(t)$, which decreases gradually over time, capturing the slow recovery or mortality dynamics associated with chronic HBV infection. The ANN closely mimics this trajectory, indicating strong generalization performance. Similarly, the susceptible male population $\textbf{S}_m(t)$, shown in Fig. 7d, follows the same exponential growth trend as females, emphasizing demographic symmetry under equal exposure assumptions. Again, the ANN prediction maintains excellent agreement. In Fig. 7e, the infected male population $\textbf{I}_m(t)$ mirrors the female infection dynamics, with a steep initial drop followed by stabilization at low levels. This behavior is well captured by the ANN with small and rapidly diminishing prediction errors. The chronically infected male population $\textbf{C}_m(t)$, shown in Fig. 7f, also exhibits a slow decline consistent with the chronic progression of HBV, and the ANN model successfully approximates this behavior. Finally, Fig. 7g presents the recovered class $\textbf{R}(t)$, which increases almost linearly as recovered individuals accumulate over time. The ANN prediction remains closely aligned with the ODE solution, and the residual error remains small throughout the time horizon.

Figures 8 and 9 illustrate the performance of the proposed ANN–LMB model in both the function fitting phase and during training diagnostics. Each subfigure in Figure 8 demonstrates a high-fidelity match between the true ODE-based numerical solutions and the ANN predictions across all state compartments. For instance, Figure 8a,d show that both the susceptible female and male populations ($S_f(t)$ and $S_m(t)$) increase over time and asymptotically approach a saturation level, reflecting natural herd immunity as infection pressures decline. In contrast, Figure 8b,e indicate an exponential decline in the infected compartments ($I_f(t)$ and $I_m(t)$), consistent with effective recovery and/or progression into chronic states. The chronic compartments ($C_f(t)$, $C_m(t)$) depicted in Fig. 8c,f also decrease steadily, suggesting that individuals either recover or exit due to HBV-related mortality. The recovered population $R(t)$, shown in Fig. 8g, demonstrates a nearly linear increase, confirming that the recovery rate is significant in the model’s progression. Notably, in all these subfigures, the ANN predictions overlap almost perfectly with the ODE trajectories, and the prediction error bar plots show only small and random deviations, affirming model accuracy and generalizability across training, validation, and testing phases. Figure 9 further supports these findings with diagnostic plots from the ANN training. Figure 9a shows an error histogram where most errors are centered around zero with minimal dispersion, highlighting balanced prediction performance across data splits. In Figure 9b, the MSE plot shows a sharp drop in error within the first few hundred epochs, with best validation performance achieved at epoch 511, demonstrating rapid and stable convergence. Figure 9c tracks optimization indicators such as the gradient norm, learning rate $\mu$, and validation checks, all of which confirm smooth training without overfitting. Finally, Figure 9d presents regression analyses, with all subsets, training, validation, test, and combined, yielding correlation coefficients $R \approx 1$. This signifies that the ANN–LMB model is both highly predictive and robust, faithfully capturing the nonlinear HBV dynamics governed by the original system of differential equations.

## Limitations

While the proposed ANN–LMB framework successfully reproduces the nonlinear dynamics of the HBV model and captures the epidemiological influence of disability parameters, several limitations should be acknowledged.

1. The model relies on parameter assumptions and literature-based estimates that may differ across populations and epidemiological settings.
2. The ANN was trained exclusively on synthetic data generated by the Runge–Kutta solver. While this ensures numerical precision, it does not account for noise, reporting errors, or incomplete observations typical of real surveillance data.
3. The current analysis assumes homogeneous mixing within gender-defined subpopulations and does not explicitly incorporate stochastic variability or age-structured heterogeneity.
4. The present implementation serves as a computational surrogate for the mechanistic model rather than a forecasting tool for real epidemics.

These factors should be considered when interpreting the results. Future extensions will focus on integrating empirical datasets and incorporating demographic and stochastic components to improve real-world applicability.

## Conclusion

This study presents a novel gender-stratified mathematical model to explore the heterosexual and homosexual transmission dynamics of HBV, incorporating HBV-induced disability into a classical compartmental framework. A key contribution of this work is the inclusion of disability-related parameters $\theta _f$, $\theta _m$, and $\delta$, which capture the onset and progression of long-term functional impairments in chronic HBV carriers. This enhancement adds biological realism and enables a more comprehensive evaluation of the broader disease burden without altering the model’s structural simplicity. To improve computational efficiency, we employed an artificial neural network (ANN) trained on numerical solutions from the classical RK4 method. The ANN–LMB framework acts as a *computational surrogate* for the mechanistic HBV model, efficiently emulating system dynamics rather than forecasting real-world epidemics. By approximating the numerical solutions of the differential equations, the ANN accelerates parameter exploration and sensitivity analysis while preserving the model’s epidemiological integrity. Comparative results confirm that the ANN closely replicates the RK4 outputs with minimal approximation error. Sensitivity analysis of the basic reproduction number ($R_0$) identifies key transmission drivers such as recruitment rates ($A_f$, $A_m$), sexual transmission probabilities ($\beta _{mm}$, $\beta _{ff}$), and contact rates ($c_{mm}$, $c_{ff}$). Mitigating factors, including vaccination rate ($\gamma _3$), disease progression rates ($\gamma _1$, $\gamma _2$), and disability-related parameters $\theta _f$ and $\delta$, are shown to suppress $R_0$ significantly. Interestingly, $\theta _m$ exhibits a mild positive influence on $R_0$, potentially reflecting behavioral asymmetries in male carriers prior to functional decline. Graphical analyses highlight the nonlinear interplay between these parameters and reveal that incorporating disability unveils a hidden epidemiological burden not captured in traditional models. Future extensions of this work will focus on coupling the ANN–LMB surrogate with surveillance or cohort-based HBV data to evaluate real-world performance and enhance predictive generalization.

## Data Availability

All data generated or analyzed during this study are included in this article.
